# Effectiveness of mHealth-Based Gamified Interventions on Physical Activity in Older Adults: Systematic Review

**DOI:** 10.2196/78686

**Published:** 2025-10-31

**Authors:** Lin Chen, Fenglin Jang, Min Li, Wei Zong, Huiqin Yu

**Affiliations:** 1 Science and Education Training Department, Pengzhou City People's Hospital, No. 255, Section 2, South Third Ring Road, Zhihe Town, Pengzhou City, Chengdu City, China, 86 13258368162; 2 School of Nursing, National University of Singapore, Singapore, Singapore; 3 Endocrinology and Metabolism Department, First Hospital of China Medical University, Shenyang, China; 4 Intensive Care Unit, Affiliated Hospital of Xuzhou Medical College, Xuzhou, China; 5 Endocrinology and Metabolism Department, Changsha People’s Hospital, Changsha, China

**Keywords:** gamification, geriatric, mobile health, older adults, physical activity, Preferred Reporting Items for Systematic Reviews and Meta-Analyses, PRISMA, systematic review

## Abstract

**Background:**

Global aging presents significant socioeconomic and health challenges, particularly for older adults who face an increased risk of chronic diseases and reduced physical activity levels. Although physical activity is crucial for maintaining health, most older adults do not meet the recommended guidelines. Gamification and mobile health (mHealth) technologies offer innovative solutions to motivate physical activity; however, research focusing on older adults is limited, especially regarding the effectiveness and sustainability of such interventions.

**Objective:**

This study aims to synthesize evidence on the effectiveness of mHealth-based gamified interventions for improving physical activity in older adults.

**Methods:**

This systematic review followed Preferred Reporting Items for Systematic Reviews and Meta-Analyses and Meta-Analysis of Observational Studies in Epidemiology guidelines and analyzed studies from PubMed, Embase, Web of Science, CINAHL, Scopus, and Wiley Online Library, covering relevant literature from their inception up to May 2025. The inclusion criteria focused on gamified mHealth interventions for adults aged 60+ years, excluding serious games. Quality assessment was conducted according to the Joanna Briggs Institute standards, with data extracted on study design, gamification elements, and outcomes such as step counts and moderate-to-vigorous physical activity.

**Results:**

Of 2944 studies identified from the database search, 1454 individuals from 8 trials were included. Gamified interventions significantly increased daily step counts and time spent in moderate-to-vigorous physical activity among older adults. Goal setting and rewards were the most frequently used components, and the combined use of mobile and wearable devices offered greater flexibility and accessibility. A classification framework indicated that interventions integrating multiple gamification elements with hybrid technology systems were most effective, particularly when guided by a theoretical basis. However, the heterogeneity in study designs, small sample sizes, and lack of long-term follow-up studies limited the generalizability of the findings.

**Conclusions:**

mHealth-based gamification interventions demonstrate potential for increasing physical activity in older adults. Future interventions should consider employing multifaceted designs combining advanced gamification with hybrid technology systems, while also prioritizing theoretical integration, long-term sustainability, and caregiver involvement to improve sustainability and inclusivity. This review highlights the need for theory-driven, technology-mediated strategies that address the unique health needs of older adults.

## Introduction

The issue of global aging is becoming increasingly pronounced. According to United Nations statistics, by 2050, the global population aged 60 years and older is projected to reach 2.1 billion, accounting for 22% of the total global population [1]. The aging of the population not only has a profound impact on the socioeconomic landscape but also presents substantial challenges in the health management of older adults. As individuals age, they commonly experience rising rates of chronic diseases, declines in physical function, and reductions in cognitive abilities—factors that collectively affect their quality of life and capacity for self-care [2].

Research indicates that daily physical activity plays a crucial role in maintaining the physical and mental health of older adults. Moderate exercise can significantly reduce the risk of chronic diseases such as cardiovascular disease, diabetes, and osteoporosis, while also promoting cognitive function and mental well-being [3]. However, the reality is that the daily activity levels of most older adults fall far below the recommended health guidelines. A sedentary lifestyle has become a prevalent issue, and insufficient physical activity accelerates functional decline, significantly increasing the risk of falls, disability, and social isolation [4]. Thus, identifying effective strategies to enhance the physical activity levels of older adults is a critical issue that requires urgent attention in public health.

Gamification, as an innovative behavioral intervention strategy, has garnered increasing attention in health promotion in recent years. Gamification involves incorporating game design elements—such as points, leaderboards, progress bars, and badges—into nongame contexts to motivate and enhance positive user behavior [5]. Existing studies have shown that gamification-based interventions are highly effective in promoting health behaviors, rehabilitation training, and chronic disease management, significantly improving participants’ motivation and adherence [6-8].

Mobile health (mHealth) refers to a comprehensive technological system that employs mobile devices, sensors, and digital media technologies for health monitoring, disease management, and health promotion [9]. Compared to traditional gamified interventions, mHealth gamified interventions provide remote health management and real-time feedback and integrate personalized, adaptable health content. This enables older adults to engage in health activities more flexibly within their daily routines. Moreover, the widespread use of mobile devices allows this intervention model to reach a broader population of older adults, particularly in home care and telemedicine settings, where its advantages are particularly pronounced [10].

Although gamified interventions combined with mHealth technology show considerable potential in enhancing the activity levels of older adults, challenges such as cognitive decline, lower education levels, and limited digital proficiency among older adults have hindered their broader adoption [11,12]. As a rapidly growing group of technology users, older adults have unique needs and face significant challenges, but they are expected to undergo a phase of rapid development in the near future. However, there is currently a lack of systematic quantitative research that comprehensively evaluates the actual effects of gamified interventions on the physical functioning and activity levels of older adults. Therefore, synthesizing existing empirical studies into a systematic review to clarify their effectiveness and applicability holds significant academic and practical value.

Specifically, this study focuses on the following core issues:

1. Does gamification based on mHealth offer advantages over traditional interventions in promoting physical activity in older adults?
2. What are the most frequently used gamification elements and mHealth types in the existing studies?
3. Is the process of gamification design integrated with behavioral or other related theories or principles?
4. What factors may affect the feasibility and effectiveness of gamified interventions for older adults?

## Methods

### Overview

The reviewed protocol was developed and approved by all authors prior to initiation. It complies with all recommendations from the systematic review, strictly adhering to the PRISMA (Preferred Reporting Items for Systematic Reviews and Meta-Analyses) checklist and Meta-Analysis of Observational Studies in Epidemiology guidelines [13]. Literature searches were performed across the following databases: PubMed, Web of Science, Embase, CINAHL, Scopus, and Wiley Online Library. The search period spanned from each database’s inception until May 20, 2025. This study was registered with the International Prospective Register of Systematic Reviews (PROSPERO; registration number CRD420251056689).

### Search Strategy

In most studies on gamification, younger individuals are typically the primary target group for game-based interventions due to their generally higher sense of self-efficacy and greater experience with digital gaming. In this context, the study initially focused on the research subjects and intervention measures but overlooked other retrieval elements when designing the retrieval strategy. However, during the subsequent literature screening, these overlooked elements will be reassessed and evaluated. To maximize the breadth of the search, the study selected the keywords “gamification,” “game elements,” and “elderly” and refined the retrieval strategy by incorporating a combination of controlled vocabulary, natural language terms, and synonyms, in accordance with the requirements of different databases. An additional file shows the search strategy in more detail (Multimedia Appendix 1).

### Literature Inclusion Criteria

To ensure that the analyzed literature aligns with the objectives and to mitigate the impact of conceptual confusion on the validity of the research results, this review clarifies the distinction between serious games and gamification, incorporating this differentiation throughout the literature selection process. Gamification refers to the integration of game elements, such as points, leaderboards, and progress bars, into nongame environments to enhance user engagement and motivation through game-like experiences, without altering the nature of the tasks. For example, in rehabilitation training for older adults, adding a reward system or virtual progress bar can extend training time [14].

Serious games, on the other hand, represent a comprehensive design approach that provides an immersive gaming experience, with the primary goal of education, training, or therapy, rather than entertainment. They are sometimes referred to as purpose-driven games (eg, virtual reality rehabilitation training games for older adults) [15,16]. However, the distinction between these 2 concepts may be ambiguous and subjective. When these concepts cannot be clearly differentiated, the research team engages in discussion and makes a final decision.

The studies included in this systematic review must meet the following criteria: (1) studies targeting older adults with a mean age ≥60 years and minimum age threshold aligned with World Health Organization functional aging criteria (typically ≥55 y). When the exact minimum age was unreported, statistical estimation (mean − 2SD) confirmed ≥90% of the participants met age criteria. (2) The use of gamification or at least 1 game element is clearly specified. Serious games (eg, video games, immersive virtual reality games, and augmented reality games) or studies that merely mention gamification without analysis are excluded. (3) Gamification is implemented through digital devices such as tablets, smartphones, or wearables. (4) At least 1 change related to physical or social activities is described, which may be presented as subjective self-reports or objective measurement indicators. (5) The study is original empirical research (must be experimental in nature). Review articles, research proposals, guidelines, policy documents, and research programs are excluded. (6) Only peer-reviewed, published papers in English are included, including doctoral dissertations and conference papers.

### Quality Assessment

The research team conducted quality assessments of the studies that met the inclusion criteria, following the quality assessment standards established by the Joanna Briggs Institute [17,18]. There are 13 criteria for evaluating randomized controlled trials (RCTs) and 9 criteria for evaluating quasi-experimental studies. Studies that meet at least 50% of the assessment criteria are accepted for review. For RCTs, the research team decided to exclude the blinding of researchers and participants from the quality assessment, as blinding is considered impractical in the context of gamified interventions. Any disputes are resolved through discussions with a third-party investigator to reach a final consensus.

### Data Extraction and Analysis

Two researchers independently extracted data from the selected studies using standardized forms based on the content of the literature. The extracted data included the first author’s name, publication year, country, study design, subject characteristics (sample size, mean age, and gender distribution), intervention characteristics (study setting, type of equipment, and duration), gamification characteristics (game name, game elements, and theories used), primary and secondary outcomes related to physical activity, and a summary of the research conclusions. Any disputes were resolved through discussions with a third-party investigator to reach a final consensus.

Due to substantial methodological and reporting differences that prevented quantitative data synthesis, we conducted a narrative synthesis organized by the PICO framework to systematically summarize the findings of the studies.

To systematically address heterogeneity in gamification elements and technological platforms, we developed a dual-axis classification framework. The first dimension categorizes interventions by gamification complexity, distinguishing basic designs employing 1 or 2 core game elements from intermediate configurations integrating 3 or 4 elements, and advanced systems incorporating 5 or more interactive components. The second dimension classifies technological integration into standalone mobile apps, hybrid systems combining mobile apps with wearable devices, and wearable-centric approaches relying primarily on sensor technology. This framework enables structured cross-study comparisons while acknowledging inherent methodological variations.

## Results

### Search Outcomes

**Figure 1.**
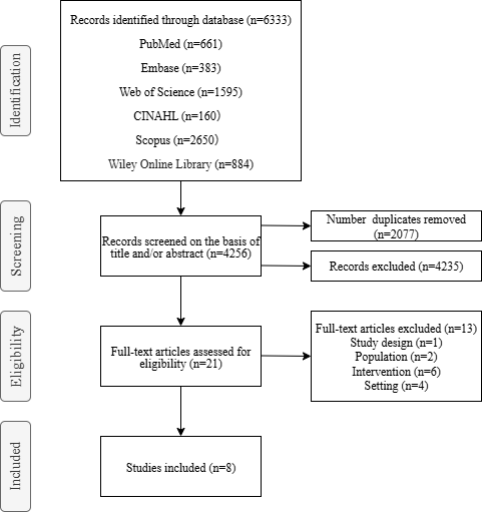
PRISMA (Preferred Reporting Items for Systematic Reviews and Meta-Analyses) flow chart.

A total of 6333 records were identified. After removing 2077 duplicate entries using EndNote, the remaining 4256 records were screened by title and abstract. Following rigorous assessment, a substantial number of records were excluded, primarily due to ineligible age, nonphysical activity focus, or the others (Multimedia Appendix 2). Subsequently, 21 articles were initially included. Reviews, clinical record comparisons, case reports, and other literature that did not meet the inclusion criteria were excluded based on their titles and abstracts. Following a more detailed examination of the full text, 13 articles were further excluded for not meeting the inclusion criteria. An additional file shows this in more detail (Multimedia Appendix 3). Ultimately, 8 studies [14,19-25] were included in this review. The search results are presented in Figure 1.

### Study Characteristics

The studies selected for this review were conducted between 2018 and 2024 in Sweden [14], Portugal [19], Japan [20,21,24], Brazil [21], and the United States [23,25]. At least 5 studies [14,22-25] were randomized controlled trials, while 3 studies [19-21] were quasi-experimental studies. Sample sizes ranged from 12 to 1062 participants, with a total of 1454 participants. The average age of participants ranged from 62 to 81.5 years, with a higher percentage of women. At least 4 studies [20-22,24] included community-dwelling older adults, 1 study [14] included participants with musculoskeletal conditions, 1 study [23] included participants at risk of cardiovascular disease, 1 study [25] included participants at risk of Alzheimer disease, and 1 study [19] did not specify participant characteristics or the gender ratio. A summary of the participants, measures, interventions, controls, and outcomes is presented in Table 1.

**Table 1.** Study characteristics.

Authors/year/country	Duration /study design	Participants	Gamification characteristics	Intervention(s)	Results	Conclusions
		Characteristics/age/sample size (female, n, %)	Game name/elements/theory used	Sample/program/mobile device		
Randriambelonoro et al [14] 2023 (Switzerland)	RCT^a^ 3-week intervention +3-week follow-up period	Older adults with musculoskeletal problems Mean (SD) age: 81.5 (6.8) y Sample size: 57 (39, 68.4%)	Name: activLife Elements: challenges/customization/goals/progress bars Theory: none	IG^b^ (35): 3 times/week gamified rehabilitation training using Stepwatch activity monitor combined with ActivLife device CG^c^ (22): standard rehabilitation treatments	Steps were slightly higher but not significantly different in IG (IG:1839 vs CG:1504 steps, *P*=.25)	Game-based rehabilitation training is as effective as standard care in restoring functional ability and has a promoting effect on daily activity
Martinho et al [19] 2023 (Portugal)	Single-group (pre-post) study 4-week baseline +1-week intervention period	older adults aged 55 years and above Mean age: 62.0 y Sample size: 12 (no data)	Name: coaFEId Elements: levels/challenges/rewards/feedback/customization/progress bars Theory: circumplex model of affect (based on the dimensions of wakefulness and valence) [26]	IG(12): coaFEId application installed on smartphones	Significant difference in daily steps between pre-post intervention (2532 vs 4931 steps, Cohen *d*=0.65, *P*=.03)	Game-based agent system significantly improved daily steps of older adults in the short term and had high availability
Santos et al [20] 2018 (Japan)	Single-group (pre-post) study 1-week baseline +2-week intervention period	Older adults in communities Mean (SD) age: 75.0 (3.4) y Sample size: 12 (9, 75%)	Name: shinpo Elements: rewards/challenges/collaboration/goals/avatars Theory: none	IG(9): collecting cards by smartphones that are preinstalled with Shinpo	7.6%‐8.1% more steps than the baseline (mean: 22,567.2, all participants)	Gamification improves the activities of older adults, but the mechanism of social interaction needs to be optimized
Santos et al [21] 2019 (Brazil)	Quasi-experimental Study 4-week intervention period	Older adults in communities Mean (SD) age: 62.3 (5.8) y Sample size: 18 (14, 78%)	Name: trilhas Elements: rewards/challenges/goals/social support/collaboration Theory: none	IG(11): trilhas application installed on smartphones with social interaction function CG(7): trilhas application installed on smartphones without social interaction function	Step counts significantly increased in IG (absolute change: *η*²=0.19, *P*=.01; percent change: *η*²=0.27, *P*=.009)	Social interaction in gamification can effectively improve the level of physical activity in older adults
Santos et al [22] 2021 (Japan)	RCT 1-week baseline +3-week intervention period	Older adults in communities Mean (SD) age: 63.4 (6.8) y Sample size: 18 (14, 78%)	Name: shinpo Elements: rewards/goals/collaboration/social support Theory: None	IG(9): shinpo application installed on smartphones with social interaction function to collect cards CG(9): shinpo application installed on smartphones without social interaction function to collect cards	Step counts significantly increased in IG (absolute change: *η*²=0.31, *P*=.04; percent change: *η*²=0.30, *P*=.03)	Social interaction in gamification can effectively improve the level of physical activity in older adults
Fanaroff et al [23] 2024 (America)	RCT 12-month intervention +6-month follow-up period	Older adults with high risk of cardiovascular Mean (SD) age: 67 (8.0) y Sample size: 1062 (648, 61%)	Name: none Elements: levels/goals/points/social support/rewards Theory: prospect theory [27]	G1(304): gamified intervention based on Fitbit Charge (wearable device)/smartphone/tablet G2(302): financial incentive intervention based on Fitbit Charge (wearable device)/smartphone/tablet G3(305): gamification+financial incentives CG(151): feedback only	Significant difference in daily steps (538 steps, *P*=.003; and follow-up 459.8 steps, *P*=.017) and MVPA (min/wk) (15.4, *P*=.033) between G1 and CG	Gamification significantly improved the activity of older adults and was more suitable for clinical promotion because of its lower cost.
Kawaguchi et al [24] 2024 (Japan)	RCT 12-week intervention period	Older adults in communities Mean (SD) age: 70.0 (6.3) y Sample size: 181 (100, 55.3%)	Name: ESP Elements: levels/feedback Theory: behavior change [28]	IG(87): ESP application installed on smartphones combined with Google Fit CG(94): Google Fit only	Significant difference in activity frequency (times/2 mo) (3.03, *P*=.04) but not in weekly steps	ESP significantly improved the activity of older adults but was ineffective for step counts
Greysen et al [25] 2024 (America)	RCT 12-week intervention +3-week follow-up period	Older adults at risk of Alzheimer disease Mean age: 70.6 (3.1) y Sample size: 94 (73, 78%)	Name: none Elements: rewards/levels/goals/feedback/collaboration Theory: prospect theory [27]	IG(44): text messages–based gamification and social support combined with FitBit device/application CG(50): FitBit device/application only	Significant difference in daily steps (*Δ*=1699 steps, *P*<.0001) and MVPA (min/day) (*Δ*=6.6, *P*=.017 between 2 groups	Gamification combined with supportive partners can effectively increase physical activity in high-risk older adults, and the effect is sustainable

a RCT: randomized controlled trial.

b IG: intervention group.

c CG: control group.

### Risk of Bias

The quality assessment scores for the RCT studies ranged from 7 to 10 points (Table 2). All 5 studies employed randomization; however, the concealment of treatment group allocation was unclear in 3 studies. Due to the nature of the intervention, blinding of participants or personnel was not possible. In 3 studies [23-25], outcome assessors were blinded to the treatment allocation. Control of confounding factors was unclear in 1 study [14]. The average score was 9/11, indicating that all the RCT studies demonstrated high methodological quality.

The 3 quasi-experimental studies each received a score of 6 points (Table 3) for methodological quality. In 1 study [21], the age of participants in the 2 groups was not similar, and the characteristics of health status and technical proficiency were not clearly presented. The criteria for this assessment were not explicit. None of the studies reported in detail whether participants in the intervention group received any other care or treatments besides the intervention. At least 2 studies [19,20] employed before-and-after designs without an independent control group. Another 2 studies [20,21] had incomplete follow-ups, and although the reasons for dropout were stated, the impact of dropout on the results was not analyzed. At least 1 study [20] only reported descriptive statistics (means, standard deviations) and did not perform significance testing or calculate effect sizes. The statistical methods used were relatively simple.

**Table 2.** Quality assessment scores of the selected RCT studies^a^.

Authors (year)	1	2	3	4	5	6	7	8	9	10	11	12	13	Total
Randriambelonoro et al [14] 2023	Y	N	Y	N/A	N/A	?	?	Y	N	Y	Y	Y	Y	7/11
Santos et al [20] 2018	Y	?	Y	N/A	N/A	?	Y	Y	Y	Y	Y	Y	Y	9/11
Fanaroff et al [23] 2024	Y	?	Y	N/A	N/A	Y	Y	Y	Y	Y	Y	Y	Y	10/11
Kawaguchi et al [24] 2024	Y	Y	Y	N/A	N/A	Y	Y	Y	Y	Y	N	Y	Y	10/11
Greysen et al [25] 2024	Y	Y	Y	N/A	N/A	Y	Y	Y	N	Y	Y	Y	Y	10/11

a Rating scale: Yes (Y), No (N), Unclear (?), Not applicable (N/A); items for critical appraisal: 1. Were the participants' group assignments sufficiently randomized? 2. Was the control group's allocation concealed? 3. At baseline, were the groups comparable? 4. Were staff members and participants blinded to the intervention’s allocation? 5. Were the researchers blinded to the allocation of the intervention? 6. Did the outcome assessors have blinded access to the allocation of the intervention? 7. Aside from the intervention, did the control group receive the same care as the intervention group? 8. Was the follow-up completed? If not, were group differences sufficiently explained and analyzed? 9. Were participants assessed with the goal of randomization in mind? 10. Did all intervention groups employ the same techniques for measuring outcomes? 11. Were the outcome measurements accurate? 12. Was the correct statistical analysis applied? 13. Was the trial design suitable? Did the analysis and interpretation account for any deviations from the conventional RCT design?

**Table 3.** Quality assessment scores of the selected quasi-experimental studies^a^.

Authors (year)	1	2	3	4	5	6	7	8	9	Total
Martinho et al [19] 2023	Y	Y	?	N	Y	N	Y	Y	Y	6/9
Santos et al [21] 2019	Y	Y	?	N	Y	Y	Y	Y	N	6/9
Santos et al [22] 2021	Y	N	?	Y	Y	N	Y	Y	Y	6/9

a Rating scale: Yes (Y), No (N), Unclear (?), Not applicable (NA); items for critical appraisal: (1) Are the study’s “cause” and “effect” (ie, the order of the variables) evident? (2) Are the individuals being compared similar? (3) Aside from the intervention, do the individuals in the comparison group receive the same care or treatment? (4) Is a control group present? (5) Are the results measured more than once before and after the intervention? (6) Has the follow-up been completed? If not, are the differences sufficiently explained and analyzed? (7) Do the individuals being compared have the same outcome measured in the same way? (8) Is the outcome measurement accurate? (9) Is the correct statistical analysis applied?

### Characteristics of the Described Interventions

Table 1 and Multimedia Appendix 4 display the gamification features of the studies included in our systematic review. The number of game elements used in the gamification interventions for older adults' physical activity ranged from 2 to 6, with the majority incorporating 5 game elements. The most commonly used game elements were goals and rewards, followed by challenges and collaboration, while virtual identity and points were the least used.

Half of the 8 studies [19,23-25] employed theories or principles to design the gamification interventions. At least 2 studies [23,25] conducted in the United States used prospect theory from behavioral economics, while the other 2 studies employed the theory of behavioral change [24] and the emotional circumplex model [19]. All of these studies used a single theory without combining them.

In 1 study, family members or friends were incorporated into the gamified intervention as support partners to provide encouragement and assist participants in achieving their activity goals.

There are 3 digital approaches to gamification for older adults. Most studies [19-22] use mobile apps only (4/8), while some studies [14,24,25] combine mobile apps with activity monitors (3/8), and only 1 study [23] uses activity monitors alone (eg, Stepwatch or Fitbit). The shortest intervention time was 1 week [19], and the longest was 12 months [23]. Only 3 studies [18,23,25] included a follow-up period, highlighting the need for further evaluation of gamification interventions to assess their long-term sustainability. Another 5 studies [14,21,22,24,25] compared a gamification intervention to usual care, while 2 studies [19,20] did not include a control group. The study by Fanaroff et al [23] assigned participants to 4 groups and compared the effects of gamification to financial incentives, a combined intervention of both, and explored the impact of gamification on physical activity in older adults.

### Effectiveness of Interventions

The included studies all assessed the effect of gamification interventions on the number of steps taken by older adults in their daily lives, as measured by activity monitors or smartphone sensors such as Google Fit. The findings from both controlled and single-arm studies were largely consistent in confirming the positive impact of gamification interventions on step count, including results observed during the follow-up period. The only exception was the study by Kawaguchi et al [24], which reported a nonsignificant increase in the number of steps following the gamification intervention. However, this study did report that intervention participants had a higher frequency of participation in specific types of activities, particularly hobbies and culture-related activities, compared to the control group.

At least 2 RCTs [23,25] reported changes in the time spent in moderate-to-vigorous physical activity (MVPA) per day, as measured by activity monitors. After adjusting for factors such as baseline activity levels, proficiency with the devices, and genetic risk, between-group differences indicated that gamification can significantly increase the duration of MVPA in older adults, with these effects being sustainable.

### Patterns From Intervention Classification

Application of the classification framework revealed consistent patterns across the 8 included studies. Advanced gamification designs consistently demonstrated superior outcomes [23,25], particularly when integrated with hybrid technology systems, showing statistically significant improvements in both step counts and MVPA duration [23,25]. Basic gamification approaches proved effective for step count enhancement [20,21] but exhibited limited capacity for tracking complex activity metrics [19]. Standalone mobile apps demonstrated strengths in facilitating social interaction mechanisms [20,22], while wearable-centric implementations may suffer from scalability limitations [14]. Crucially, all advanced interventions incorporated theoretical frameworks [19,23-25], whereas basic and intermediate designs operated predominantly without explicit theoretical foundations [20-22] (Table 4).

Effect strength was evaluated through standardized criteria: strong, >15% improvement from baseline OR Cohen *d* >0.8 OR *η*²>0.25 with *P*<.05; moderate, 5%‐15% improvement OR Cohen *d* 0.5‐0.8 OR *η*² 0.10‐0.25 with *P*<.05; weak, <5% improvement OR Cohen *d* <0.5 OR *η*² <0.10 with *P*<.05, NS, nonsignificant.

**Table 4.** Systematic categorization of interventions.

Authors (year)	Complexity	Tech integration	Primary outcome	Effect direction	Statistical significance	Theoretical framework
Randriambelonoro et al [14] 2023	Intermediate	Wearable-centric	Daily steps	NS^a^	*P*=.25	None
Martinho et al [19] 2023	Advanced	Standalone	Daily steps	Moderate	Cohen *d*=0.65 *P*=.03	Circumplex model
Santos et al [20] 2018	Basic	Standalone	% Steps	Moderate	Descriptive statistics only	None
Santos et al [21] 2019	Basic	Standalone	% Steps	Moderate	*η*²=0.19, *P*=.01	None
Santos et al [22] 2021	Intermediate	Standalone	% Steps	Moderate	*η*²=0.31, *P*=.04	None
Fanaroff et al [23] 2024	Advanced	Hybrid	Daily steps and MVPA^b^	Strong	*P*=.08^c^ *P*=.03^d^	Prospect theory
Greysen et al [25] 2024	Advanced	Hybrid	Daily steps and MVPA	Strong	*P*<.001^c^ *P*=.08^d^	Prospect theory
Kawaguchi et al [24] 2024	Intermediate	Hybrid	Weekly steps and activity frequency	Weak	*P*=.04 (steps NS)	Behavior change

a NS: nonsignificant.

b MVPA: moderate-to-vigorous physical activity.

c *P* value of daily steps.

d *P* value of MVPA.

## Discussion

### Principal Findings

The present review included only 8 full-text articles that met stringent inclusion criteria, reflecting both methodological rigor and domain-specific constraints. Strict adherence to gamification standards, mandatory mHealth components, and a focused eligibility criterion of adults aged 60 years and above resulted in the exclusion of a considerable proportion of initially identified records. This selectivity contrasts sharply with the broad application of gamification in general healthcare, which has largely targeted younger populations [11]. Nonetheless, this condensed evidence base captures a developing nexus among gerontechnology, behavioral engagement, and mHealth—an area that remains understudied yet highly relevant for physical activity promotion in older adults. Despite these constraints, the main findings indicate that mHealth-based gamified interventions offer meaningful benefits in improving physical activity levels within this demographic.

A larger sample size and longer intervention duration would enhance the generalizability of the data and provide more opportunities to determine usage patterns and preferences. However, most of the reviewed studies employed shorter intervention durations and did not include follow-up assessments, involving only a small number of participants. This phenomenon is also observed in other areas of gamified interventions for older adults. For example, Savulich et al [29] used a sample size of 21 participants in their intervention group for cognitive training using iPads for older adults diagnosed with amnestic mild cognitive impairment, with an intervention duration of 4 weeks. Costa et al [30] used a sample size of 20 participants in their intervention group for mental health interventions for community-dwelling older adults through a gamified learning platform, with an intervention duration of 6 weeks. Despite the increased opportunities to access new technologies in recent years, the application of these tools among the older adult population still faces significant challenges.

Choosing game elements that cater to the needs of older adults or developing corresponding gamified applications can significantly enhance the effectiveness, practicality, and sustainability of interventions, thereby increasing user participation and reducing sample attrition rates during follow-up processes [31]. The studies included in this review generally adopt goal-setting (eg, daily step counts) and instant rewards (eg, points and badges) as core elements. These elements are widely used due to their strong alignment with the core mechanisms of behavior change [32], while also addressing the research need for quick verification of the immediate effects of gamification through short-term interventions. This approach aligns with the conclusions of 2 previous systematic reviews, which did not specify the research subjects [33,34].

However, the adoption of complex gamification elements, such as virtual identities and social collaboration, requires older adults to construct abstract self-representations or coordinate rules for multilateral interactions. This may lead to cognitive load or operational obstacles for some older adults [29], resulting in fewer studies designed with such elements. Additionally, we note that the included studies all employed interventions with 2 or more combined elements, suggesting that combinations of multiple game elements may be more effective than single elements. However, due to the lack of detailed information in the initial studies, no definitive conclusions can yet be drawn about the optimal number or composition of game elements in these interventions.

One point that needs clarification is that the most frequently used gamification elements do not necessarily indicate the optimal effectiveness of those elements on older adults or physical activity. As Dugas et al [35] pointed out, personality differences can significantly affect how certain gamification characteristics influence older adults’ use of mobile health technologies. When gamification mechanisms are unsuitable, older adults often fail to understand and recognize the relevant benefits of these services. Therefore, we must approach the results of this systematic review with caution, as it could lead to cognitive biases. In the study by Kostopoulos et al [36], the authors established goal-setting and achievement features that allow users to receive different rewards after completing various goals and tasks. The purpose was to further explain how these goals and tasks are generated for each user, which may help in understanding the different motivations and preferences that drive users to perform various tasks. This is an innovative approach, as it provides feedback on user performance while validating the effects of gamification interventions.

We have also found that Greysen et al [25] systematically included primary caregivers (support partners) as a core component of the intervention. And although other studies did not independently analyze or evaluate the involvement of nursing staff, most of them indirectly addressed similar concepts through social functions or external support mechanisms. This suggests that incorporating nursing staff into the network could be a valuable development. Including family members, health care professionals, and social workers in mobile health gamification interventions may enhance older adults’ perceptions of social and emotional support, thereby increasing their acceptance of these interventions [37]. Additionally, from a technical perspective, this model can improve older adults’ understanding of the intervention content and their sense of security in using the interventions [38]. This approach is particularly beneficial for older adults who are interested in gamification but lack independent operational skills, as well as for those who prefer direct care from health care professionals, providing an effective pathway for the broader application of gamification.

Technological platforms that accompany gamification are another important factor attracting the interest of older adults. Currently, validated technological features used among the older adult population include self-management systems [9], physical robots [10], consoles [11], portable devices, and wearable technology [12]. Although studies by Kitakoshi et al [38] and Mocanu et al [39] have confirmed that older adults enjoy using systems and interacting with robots, mobile health offers more diverse gamification opportunities in real-world applications. This aligns with the viewpoint of Steinert et al [40], who pointed out that older adults are more motivated by tangible information provided by activity monitors, sensors, and smartphones, rather than more abstract game feedback. This is also the primary reason why our review is limited to gamification intervention research supported by mobile technology.

In this systematic review, the mHealth technologies used to provide physical activity gamification interventions include 1 activity monitor (1/8), 4 mobile apps (4/8), and 3 combinations of both (3/8). This distribution of technological application patterns reflects a shift in the current research field, from single-device use to multimodal and collaborative intervention plans. Tablet devices offer significant advantages in screen size and clarity, and previous research [30] has confirmed their ability to improve specific cognitive functions in older adults. However, the physical characteristics of the devices, such as weight, button design, and keyboard operation, may affect the user experience of older adults participating in activities. As a result, only 1 study in this review mentioned tablet devices [23].

The theoretical framework also warrants discussion, as it enables a deeper analysis of the behavioral mechanisms behind older adults’ participation in physical activities, accurately addressing their physiological, psychological, and social needs. However, among the reviewed studies, only 4 [19,23-25] explicitly adopted a single theoretical framework or model to design gamified interventions for physical activities targeting older adults. The classification framework revealed that these theory-oriented designs demonstrated more effective outcomes while accommodating greater gamification complexity, suggesting that theoretically integrated gamification may play a positive role in reducing engagement burden among older adults.

At least 2 of these studies [23,25] utilized behavioral economics, incorporating design principles such as loss aversion, precommitment, and the fresh start effect. The significance of this theoretical application lies in viewing participation in physical activities as an investment in future health, guiding older adults’ behavioral choices through subtle environmental adjustments rather than rigid directives. In another study [19], the circumplex model of affect was translated into operational gamification solutions, offering new insights for the design of digital therapeutics in geriatric health.

Self-Determination Theory, a comprehensive motivation theory, has become a key framework for health behavior interventions, but only 1 study [24] claims to have used it, differing from the 2 systematic reviews mentioned earlier [33,34]. Other commonly considered behavior change theories include the Health Belief Model [41], Theory of Planned Behavior [42], Transtheoretical Model [43], and Habit Formation Theory [44], but these theories appear to be rarely applied in gamified interventions for older adults, even in nonphysical activity areas. The reasons for this may partly stem from the relatively novel concept of introducing gamified design to the older adult population, where traditional theories may not align well. Alternatively, it could be that the behavior patterns of older adults are complex and diverse, making the application of theory more challenging. Therefore, a more systematic and appropriate gamification theory system for the older adult population could be developed in the future through qualitative exploration, theoretical integration, and other methods.

Finally, a study published by Bellotti et al [45] in the field of human-computer interaction suggested that game-based interventions could serve as a strategy for patient self-assessment, enriching the typical content of assessments for older adults. Although no authors in the reviewed studies directly made such a claim, this idea may also apply to gamification for older adults. By continuously tracking the activity performance of older adults, research can gather multidimensional data, such as task completion rates, execution times, and failure counts. This information, when combined with commonly used assessment tools for older adults, such as the Activity of Daily Living scale [46] and the Morse fall risk assessment scale [47], could provide a more comprehensive reflection of functionality and psychological state, thereby guiding clinical decision-making and personalized intervention strategies. While this is a reasonable inference, further investigation is needed. Additionally, it is important to consider data privacy and the risk of false positives to avoid excessive monitoring, which could lead to psychological stress or self-report biases due to the Hawthorne effect.

### Limitations

Although this study systematically reviews the effectiveness of mHealth gamification interventions on physical activity in older adults, several limitations should be noted. First, the included studies are relatively few (n=8), with generally small sample sizes, potentially limiting the generalizability and reliability of the findings. Second, intervention durations varied significantly (1 wk to 12 mo), and most studies lacked follow-up periods, complicating the assessment of long-term sustainability. Additionally, considerable heterogeneity existed across studies, including variations in gamification elements, technological platforms, theoretical frameworks, and outcome measurement methods, precluding meta-analysis and unified conclusions. Another limitation is the incomplete reporting of baseline participant characteristics (eg, digital literacy, health status), potentially biasing intervention effect interpretations. Finally, as most studies focused on high-income countries (eg, the United States, Japan, Switzerland), the findings may lack generalizability to older adults in other regions or cultural contexts. Furthermore, due to incomplete reporting of minimum participant ages, this review inferred population homogeneity using a functionally oriented aging definition and statistical estimation, which may overestimate or misrepresent intervention effectiveness.

### Conclusions

This systematic review suggests that mHealth-based gamified interventions have potential benefits in increasing the physical activity levels of older adults, particularly by enhancing daily step counts and the duration of MVPA. Common gamification elements, such as goal-setting and immediate rewards, effectively stimulate the participation motivation of older adults, while the combination of mobile apps and wearable devices further enhances the flexibility and accessibility of these interventions. However, existing evidence still has limitations, including a limited number of studies, small sample sizes, and a lack of long-term follow-up data. Future studies should aim to expand sample sizes, extend intervention durations, and include more diverse older adult populations to validate the universality and sustainability of the intervention effects. Additionally, there is a need to further explore the optimal combination of gamification elements, the integration of theoretical frameworks, and the potential impact of caregiver network participation on the effectiveness of these interventions. Overall, mHealth gamified interventions offer an innovative and feasible strategy for promoting physical activity among older adults, but their design and implementation must carefully consider the specific needs and varying abilities of older adults to ensure the practicality and acceptance of these interventions.

## Supplementary material

10.2196/78686 Multimedia Appendix 1 Full search strategy.

10.2196/78686 Multimedia Appendix 2 References screened by title and abstract.

10.2196/78686 Multimedia Appendix 3 References excluded after reading the full text.

10.2196/78686 Multimedia Appendix 4 Game elements observed in the studies.

10.2196/78686 Checklist 1 PRISMA checklist.

## Abbreviations

mHealth: mobile health
MVPA: moderate-to-vigorous physical activity
PRISMA: Preferred Reporting Items for Systematic Reviews and Meta-Analyses
RCT: randomized controlled trial
