# Supplementary material for: Effectiveness of mHealth-Based Gamified Interventions on Physical Activity in Older Adults: Systematic Review
Source: JMIR Aging. 2025 Oct 31;8:e78686. doi: 10.2196/78686 (PMC12577663; doi:10.2196/78686)
Supplement: Multimedia Appendix 1 [file aging-v8-e78686-s001.docx]

**Multimedia Appendix 1. Full search strategy.**

We employed for each database full and truncated search terms in the following search string:

Gamification: (gamif* OR “game design element*” OR “game-design element*” OR “game element*” OR “game design interface pattern*” OR “game-design interface pattern*” OR“game interface element*” OR “game mechanic*” OR “game feature*” OR “game-like element*” OR “game-like feature*” OR “videogame element*”) AND aged:（（aged OR “ageing” OR “aging” OR “elder*” OR “older adult*” OR “older person*” OR “older people” OR “senior*” OR “senior citizen*” OR “geriatric*” OR “retired” OR “retiree*” OR “pensioner*”））

Although the search strategy was the same for each database, suitable changes were made to accommodate for the different interfaces.

**Pubmed Hits:661**

（(gamif* OR “game design element*” OR “game-design element*” OR “game element*” OR “game design interface pattern*” OR “game-design interface pattern*” OR“game interface element*” OR “game mechanic*” OR “game feature*” OR “game-like element*” OR “game-like feature*” OR “videogame element*”) AND（aged OR “ageing” OR “aging” OR “elder*” OR “older adult*” OR “older person*” OR “older people” OR “senior*” OR “senior citizen*” OR “geriatric*” OR “retired” OR “retiree*” OR “pensioner*”））

**Embase Hits:383**

(‘gamif*':ti,ab,kw OR ‘game design element':ti,ab,kw OR ‘game-design element':ti,ab,kw OR ‘game element':ti,ab,kw OR ‘game design interface pattern':ti,ab,kw OR ‘game-design interface pattern':ti,ab,kw OR ‘game interface element':ti,ab,kw OR ‘game mechanic':ti,ab,kw OR ‘game feature':ti,ab,kw OR ‘game-like element':ti,ab,kw OR ‘game-like feature':ti,ab,kw OR ‘videogame element':ti,ab,kw) AND (‘aged':ti,ab,kw OR ‘ageing':ti,ab,kw OR ‘aging':ti,ab,kw OR ‘elder*':ti,ab,kw OR ‘older adult*':ti,ab,kw OR ‘older person*':ti,ab,kw OR ‘older people':ti,ab,kw OR ‘senior*':ti,ab,kw OR ‘senior citizen*':ti,ab,kw OR ‘geriatric*':ti,ab,kw OR ‘retired':ti,ab,kw OR ‘retiree*':ti,ab,kw OR ‘pensioner*':ti,ab,kw)

**Web of Science Hits:1595**

TS=(（aged OR “ageing” OR “aging” OR “elder*” OR “older adult*” OR “older person*” OR “older people” OR “senior*” OR “senior citizen*” OR “geriatric*” OR “retired” OR “retiree*” OR “pensioner*”）) AND TS=((gamif* OR “game design element*” OR “game-design element*” OR “game element*” OR “game design interface pattern*” OR “game-design interface pattern*” OR“game interface element*” OR “game mechanic*” OR “game feature*” OR “game-like element*” OR “game-like feature*” OR “videogame element*”))

**CINAHL Hits:160**

gamification OR game design element OR game-design element OR game element OR game design interface pattern OR game-design interface pattern OR game interface element OR game mechanic OR game feature OR game-like element OR game-like feature OR videogame element AND aged OR ageing OR aging OR elder OR older adult OR older person OR older people OR senior OR senior citizen OR geriatric OR retired OR retiree OR pensioner

**Scopus Hits:2650**

( ( TITLE-ABS-KEY ( gamification ) OR TITLE-ABS-KEY ( game AND design AND element ) OR TITLE-ABS-KEY ( game-design AND element ) OR TITLE-ABS-KEY ( game AND element ) OR TITLE-ABS-KEY ( game AND design AND interface AND pattern ) OR TITLE-ABS-KEY ( game-design AND interface AND pattern ) OR TITLE-ABS-KEY ( game AND interface AND element ) OR TITLE-ABS-KEY ( game AND mechanic ) OR TITLE-ABS-KEY ( game AND feature ) OR TITLE-ABS-KEY ( game-like AND element ) OR TITLE-ABS-KEY ( game-like AND feature ) OR TITLE-ABS-KEY ( videogame AND element ) ) ) AND ( ( TITLE-ABS-KEY ( aged ) OR TITLE-ABS-KEY ( ageing ) OR TITLE-ABS-KEY ( aging ) OR TITLE-ABS-KEY ( elder ) OR TITLE-ABS-KEY ( older AND adult ) OR TITLE-ABS-KEY ( older AND person ) OR TITLE-ABS-KEY ( older AND people ) OR TITLE-ABS-KEY ( senior ) OR TITLE-ABS-KEY ( senior AND citizen ) OR TITLE-ABS-KEY ( geriatric ) OR TITLE-ABS-KEY ( retired ) OR TITLE-ABS-KEY ( retiree ) OR TITLE-ABS-KEY ( pensioner ) ) )

**Wiley online library Hits:884**

"(gamif* OR “game design element*” OR “game-design element*” OR “game element*” OR “game design interface pattern*” OR “game-design interface pattern*” OR“game interface element*” OR “game mechanic*” OR “game feature*” OR “game-like element*” OR “game-like feature*” OR “videogame element*”)" anywhere and "（aged OR “ageing” OR “aging” OR “elder*” OR “older adult*” OR “older person*” OR “older people” OR “senior*” OR “senior citizen*” OR “geriatric*” OR “retired” OR “retiree*” OR “pensioner*”）" anywhere
