# Supplementary material for: Effectiveness of mHealth-Based Gamified Interventions on Physical Activity in Older Adults: Systematic Review
Source: JMIR Aging. 2025 Oct 31;8:e78686. doi: 10.2196/78686 (PMC12577663; doi:10.2196/78686)
Supplement: Multimedia Appendix 3 [file aging-v8-e78686-s003.docx]

Multimedia Appendix 3. References excluded after reading the full text

| Authors | Title | Reasons for exclusion |
| --- | --- | --- |
| Faria,et al^[1]^. | Development and Validation of a Game for Older Adults on Lifestyles and Frailty | Serious game |
| Hurmuz,et al^[2]^. | Game not over: Explaining older adults’ use and intention to continue using a gamiﬁed eHealth service | Outcome indicators do not include PA |
| Jurivich,et al^[3]^. | A geriatric game to strengthen older adult healthcare delivery and outcomes | Outcome indicators do not include PA |
| Kooij,et al^[4]^. | Gamification as a Sustainable Source of Enjoyment During Balance and Gait Exercises | Not combined with mobile devices |
| García,et al^[5]^. | Gamification and Immersive Experiences: A Gamified Approach for Promoting Active Aging | Non-empirical research |
| Lee,et al^[6]^. | Usability of a new digital walking program  for older adults: a pilot study | Outcome indicators do not include PA |
| Pereira,et al^[7]^. | Impact of game mode in multi-user serious games for upper limb rehabilitation: a within-person randomized trial on engagement and social involvement | Serious game |
| Kappen,et al^[8]^. | Adaptive Engagement of Older Adults’ Fitness through Gamification | Outcome indicators do not include PA |
| Kamnardsiri,et al^[9]^. | Home-Based, Low-Intensity, Gamification-Based, Interactive Physical-Cognitive Training for Older Adults Using the ADDIE Model: Design,Development,and Evaluation of User Experience | Not combined with mobile devices |
| Tuan,et al^[10]^. | Assessing the Clinical Effectiveness of an Exergame-Based Exercise Training Program Using Ring Fit Adventure to Prevent and Postpone Frailty and Sarcopenia Among Older Adults in Rural Long-Term Care Facilities: Randomized Controlled Trial | Serious game |
| Xu,et al^[11]^. | Smartphone-based gamification intervention to increase physical activity participation among patients with coronary heart disease: A randomized controlled trial | Non-elderly (the average age of the participants was 53) |
| Lara,et al^[12]^. | Effects of game-based interventions on functional capacity in acutely hospitalised older adults: results of an open-label non-randomised clinical trial | Unspecified technical equipment |
| Mocanu,et al^[13]^. | A Kinect Based Adaptive Exergame | Subjects were simulated elderly people. |

PA: Physical activity

References

[1] Faria, Ana Daniela Costa Alves, Maria Manuela Martins, José Alberto Laredo-Aguilera, João Manuel Garcia Ventura-Silva, and Olga Maria Pimenta Lopes Ribeiro. 2024. “Development and Validation of a Game for Older Adults on Lifestyles and Frailty.” Nursing Reports 14(3):2499–2512. [https://doi.org/10.3390/nursrep14030184](https://doi.org/10.3390/nursrep14030184). PMID: 39311192.

[2] Hurmuz, Merve Z., Sanne M. Jansen-Kosterink, Hermie J. Hermens, and Lex van Velsen. 2022. “Game Not Over: Explaining Older Adults’ Use and Intention to Continue Using a Gamified eHealth Service.” Health Informatics Journal 28 (2):14604582221106008. [https://doi.org/10.1177/14604582221106008](https://doi.org/10.1177/14604582221106008). PMID: 35653268.

[3] Jurivich, Donald A., Richard N. Van Eck, James R. Wood, Duane Snustad, Jennifer Holloway, Derek Langendoen, Michael Hughes, Boris Galynker, Scott Brewster, and Gagandeep Dhillon Manocha. 2024. “A Geriatric Game to Strengthen Older Adult Healthcare Delivery and Outcomes.” Journal of the American Geriatrics Society 72 (S3): S82–S89. [https://doi.org/10.1111/jgs.18942](https://doi.org/10.1111/jgs.18942). PMID: 38720422.

[4] van der Kooij, Katinka, Rosalie van Dijsseldonk, Mirjam van Veen, Femke Steenbrink, Chris de Weerd, and Karin E. Overvliet. 2019. “Gamification as a Sustainable Source of Enjoyment During Balance and Gait Exercises.” Frontiers in Psychology 10:294. [https://doi.org/10.3389/fpsyg.2019.00294](https://doi.org/10.3389/fpsyg.2019.00294). PMID: 30881322.

[5] Nacimiento-Garcia, Elena, Carina S. Gonzalez-Gonzalez, Lina Colombo-Ruano, and Francisco L. Gutierrez-Vela. 2024. “Gamification and Immersive Experiences: A Gamified Approach for Promoting Active Aging.” Applied Sciences 14 (23): 10880.

[6] Lee, JungA, and HeeSung Ryu. 2023. “Usability of a New Digital Walking Program for Older Adults: A Pilot Study.” BMC Geriatrics 23 (1): 193. [https://doi.org/10.1186/s12877-023-03739-y](https://doi.org/10.1186/s12877-023-03739-y). PMID: 37003966.

[7] Pereira, Filipe, Sergi Bermúdez I Badia, Rui Ornelas, and Mónica S. Cameirão. 2019. “Impact of Game Mode in Multi-User Serious Games for Upper Limb Rehabilitation: A Within-Person Randomized Trial on Engagement and Social Involvement.” Journal of NeuroEngineering and Rehabilitation 16 (1): 109. [https://doi.org/10.1186/s12984-019-0578-9](https://doi.org/10.1186/s12984-019-0578-9). PMID: 31470877.

[8] Kappen, Dennis L. 2015. “Adaptive Engagement of Older Adults’ Fitness Through Gamification.” In Proceedings of the 2015 Annual Symposium on Computer-Human Interaction in Play, 403–6.

[9] Kamnardsiri, Teerawat, Sirinun Kumfu, Piyapat Munkhetvit, Sirinun Boripuntakul, and Somporn Sungkarat. 2024. “Home-Based, Low-Intensity, Gamification-Based, Interactive Physical-Cognitive Training for Older Adults Using the ADDIE Model: Design, Development, and Evaluation of User Experience.” JMIR Serious Games 12: e59141. [https://doi.org/10.2196/59141](https://doi.org/10.2196/59141). PMID: 39470391.

[10] Tuan, Shu-Hui, Li-Hua Chang, Shun-Fa Sun, Chia-Hui Li, Guan-Bo Chen, and Yung-Jen Tsai. 2024. “Assessing the Clinical Effectiveness of an Exergame-Based Exercise Training Program Using Ring Fit Adventure to Prevent and Postpone Frailty and Sarcopenia Among Older Adults in Rural Long-Term Care Facilities: Randomized Controlled Trial.” Journal of Medical Internet Research 26: e59468. [https://doi.org/10.2196/59468](https://doi.org/10.2196/59468). PMID: 39024000.

[11] Xu, Le, Qian Tong, Xuan Zhang, Tian Yu, Xiao Lian, Tongle Yu, Maurizio Falter, et al. 2024. “Smartphone-Based Gamification Intervention to Increase Physical Activity Participation Among Patients With Coronary Heart Disease: A Randomized Controlled Trial.” Journal of Telemedicine and Telecare 30 (9): 1425–36. [https://doi.org/10.1177/1357633X221150943](https://doi.org/10.1177/1357633X221150943). PMID: 36794484.

[12] Cuevas-Lara, Cesar, Mikel L. Sáez de Asteasu, Robinson Ramírez-Vélez, Mikel Izquierdo, Fabiola Zambom-Ferraresi, Carmen Antoñanzas-Valencia, Ana Galbete, Fabiola Zambom-Ferraresi, and Nicolás Martínez-Velilla. 2022. “Effects of Game-Based Interventions on Functional Capacity in Acutely Hospitalised Older Adults: Results of an Open-Label Non-Randomised Clinical Trial.” Age and Ageing 51 (1): afab247. [https://doi.org/10.1093/ageing/afab247](https://doi.org/10.1093/ageing/afab247). PMID: 35077558.

[13] Mocanu, Ionut, Cosmin Marian, Livia Rusu, and Roxana Arba. 2016. “A Kinect Based Adaptive Exergame.” In 2016 IEEE 12th International Conference on Intelligent Computer Communication and Processing (ICCP), 117–24. IEEE.
