# Supplementary figures and images for: Effectiveness of mHealth-Based Gamified Interventions on Physical Activity in Older Adults: Systematic Review
Source: JMIR Aging. 2025 Oct 31;8:e78686. doi: 10.2196/78686 (PMC12577663; doi:10.2196/78686)

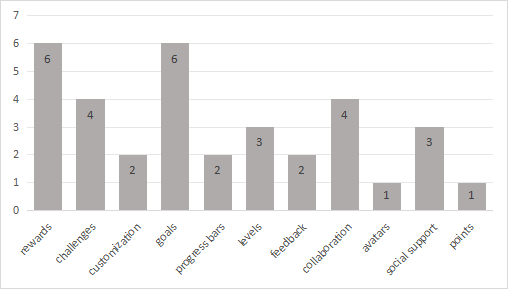

Supplement: Multimedia Appendix 4 [file aging-v8-e78686-s004.png]
